# Delays in Illness Recognition, Healthcare Seeking, and Healthcare Provision for Stillbirths and Neonatal Deaths in Seven Low- and Middle-Income Countries

**DOI:** 10.4269/ajtmh.26-0164

**Published:** 2026-10-27

**Authors:** Kitiezo Aggrey Igunza, Namrata Ramakrishna, Zachary J. Madewell, Victor Akelo, Hellen Muttai, Christopher Mugah, Paul Mitei, Ikechukwu Ogbuanu, Ima-Abasi Bassey, Erick Kaluma, Oluseyi Balogun, Ronita Luke, Shams El Arifeen, Emily S. Gurley, Mohammad Zahid Hossain, Rajib Biswas, Afsana Afrin, Nega Assefa, J. Anthony G. Scott, Haleluya Leulseged, Meron Kebede, Konjit Eshetu, Karen L. Kotloff, Samba O. Sow, Rosauro Varo, Inácio Mandomando, Marcelino Garrine, Milton Kincardett, Quique Bassat, Shabir A. Madhi, Ziyaad Dangor, Megan Dempster, Marguerite Hall, Sithembiso Velaphi, John Blevins, Maria Maixenchs, Yuting Guo, Abeed Sarker, Portia C. Mutevedzi, Cynthia G. Whitney, Chris A. Rees

**Affiliations:** ^1^ Kenya Medical Research Institute, Center for Global Health Research, Kisumu, Kenya;; ^2^ Hubert Department of Global Health, Rollins School of Public Health, Emory University, Atlanta, Georgia, USA;; ^3^ Liverpool School of Tropical Medicine, Liverpool, United Kingdom;; ^4^ Kisumu County Health Department, Kisumu, Kenya;; ^5^ The Africa Research Collaborative (The ARC), Child Health and Mortality Prevention Surveillance (CHAMPS) Programme, Freetown, Sierra Leone;; ^6^ Ministry of Health and Sanitation, Freetown, Sierra Leone;; ^7^ International Centre for Diarrhoeal Disease Research Bangladesh (icddr,b), Dhaka, Bangladesh;; ^8^ Department of Epidemiology, Johns Hopkins Bloomberg School of Public Health, Baltimore, Maryland, USA;; ^9^ London School of Hygiene and Tropical Medicine, London, United Kingdom;; ^10^ College of Health and Medical Sciences, Harar, Haramaya University, Harar, Ethiopia;; ^11^ Department of Pediatrics, Center for Vaccine Development and Global Health, University of Maryland School of Medicine, Baltimore, Maryland, USA;; ^12^ Centre pour le Développement des Vaccins-Mali, Bamako, Mali;; ^13^ Research Institute Sant Joan de Déu, Hospital Sant Joan de Déu, Barcelona, Spain;; ^14^ CIBER of Epidemiology and Public Health (CIBERESP), Madrid, Spain;; ^15^ ISGlobal, Barcelona, Spain;; ^16^ Centro de Investigação em Saúde de Manhiça (CISM), Maputo, Mozambique;; ^17^ ICREA, Pg. Lluís Companys 23, 08010 Barcelona, Spain;; ^18^ Institut Clínic de Medicina I Dermatologia, Hospital Clínic de Barcelona, Barcelona, Spain;; ^19^ Facultat de Medicina i Ciències de la Salut, Universitat de Barcelona (UB), Barcelona, Spain;; ^20^ Pediatrics Department, Hospital Sant Joan de Déu, Universitat de Barcelona, Barcelona, Spain;; ^21^ Wits Infectious Diseases and Oncology Research Institute, Faculty of Health Sciences, University of the Witwatersrand, Johannesbrurg, South Africa;; ^22^ South African Medical Research Council Vaccines and Infectious Diseases Analytics Research Unit, Faculty of Health Sciences, University of the Witwatersrand, Johannesburg, South Africa;; ^23^ Department of Paediatrics and Child Health, Faculty of Health Sciences, University of the Witwatersrand, Johannesburg, South Africa;; ^24^ Emory Global Health Institute, Emory University, Atlanta, Georgia, USA;; ^25^ Department of Biomedical Informatics, Emory University School of Medicine, Atlanta, Georgia, USA;; ^26^ Task Force for Global Health, Atlanta, Georgia, USA;; ^27^ Division of Pediatric Emergency Medicine, Emory University School of Medicine, Atlanta, Georgia, USA

## Abstract

An estimated 1.9 million stillbirths and 2.3 million neonatal deaths occur globally annually, disproportionately clustered in sub-Saharan Africa and South Asia. The aim for the present study was to determine the frequency of delays in illness recognition, healthcare seeking, and healthcare receipt among stillbirths and neonatal deaths, characterize the most common delays, and describe the frequency of antenatal care visits preceding stillbirths and neonatal deaths. A descriptive analysis was conducted using data collected from 2016 to 2023 in the Child Health and Mortality Prevention Surveillance network, which prospectively studies stillbirths and child deaths in seven low- and middle-income countries. Delays in 1) illness recognition in the home or decision to seek care, 2) transportation to healthcare facilities, and 3) the provision of clinical care (e.g., timely administration of medications) in healthcare facilities were categorized according to the “Three Delays-in-Healthcare” framework. A total of 7,079 deaths (3,424 stillbirths, 3,655 neonatal deaths) were included. Among included stillbirths, 53.6% were male; the median maternal gestational age was 36 weeks (interquartile range [IQR]: 32–38 weeks). Among included neonatal deaths, 57.3% were male; the median maternal gestational age at delivery was 34 weeks (IQR: 30–38 weeks). A total of 96.8% (*N* = 3,313) of stillbirths and 97.7% (*N* = 3,571) of neonatal deaths had ≥1 delay identified, the most common being those related to the delivery of high-quality clinical care in health facilities. Nearly half of mothers of stillbirths (48%) and 41% of mothers of deceased neonates had only one to three antenatal care visits. Delays in healthcare provision were common, suggesting that improving the quality of facility-based clinical care may be the most impactful intervention for reducing perinatal mortality.

## INTRODUCTION

Despite massive reductions in mortality among young children aged <5 years from the 1990s to the 2020s, declines in stillbirth and neonatal mortality rates have been more modest.^1^ Worldwide, there were an estimated 1.9 million stillbirths and ∼2.3 million neonatal deaths in 2023.^2^^,^^3^ Sub-Saharan Africa and South Asia, where 56% of all live births occur, account for >70% of global stillbirths and >80% of global neonatal deaths.^4^^,^^5^

Results from previous studies suggest that delayed decisions to seek healthcare by caregivers, difficulty accessing healthcare facilities, and suboptimal clinical care in healthcare facilities may contribute to high rates of childhood mortality in sub-Saharan Africa and South Asia.^6^^–^^9^ Many stillbirths and neonatal deaths are thought to be preventable when mothers access skilled healthcare personnel in a timely fashion.^2^^,^^3^ The “Three Delays in Healthcare” framework has previously been used to better understand social issues (i.e., identification of illness in the home and transportation to healthcare facilities) and health system issues (i.e., adequacy and quality of clinical care provided) that contribute to mortality among infants and children aged 1 to 59 months in Bangladesh and six sub-Saharan African countries.^10^ Stillbirths and neonatal deaths were not included in those analyses because of anticipated differences in healthcare-seeking for these populations, the interconnectedness of pregnancy-related illnesses with stillbirths and neonatal outcomes, and differences in health services delivery for neonates compared with young children.^11^ Insights are provided in the present study to better understand healthcare-seeking and provision factors that may contribute to persistently high rates of stillbirths and neonatal deaths in sub-Saharan Africa and South Asia.^12^^,^^13^

A detailed understanding of the most common delays encountered by caregivers of stillbirths and neonates who died has the potential to inform both social and clinical interventions tailored to prevent future stillbirths and neonatal deaths. The study objectives were 1) to determine the frequency of delays among stillbirths and neonatal deaths in the Child Health and Mortality Prevention Surveillance (CHAMPS) network, 2) to characterize the most common delays within the “Three Delays in Healthcare” framework, and 3) to describe the frequency and timing of antenatal care (ANC) visits among mothers of stillbirths and neonatal deaths because clinical care that may impact perinatal survival begins during pregnancy.

## MATERIALS AND METHODS

### Study design.

A descriptive analysis was conducted using data collected by CHAMPS, a prospective stillbirth and child mortality surveillance network.^14^^,^^15^ CHAMPS tracks stillbirths and deaths among children aged <5 years and performs postmortem testing to determine underlying, immediate, and comorbid causes of death for all enrolled cases. Additionally, CHAMPS determines if the stillbirth or neonatal death could have been prevented and provides insights into possible interventions that could have prevented the deaths.

### Study setting.

CHAMPS operates in regions with disproportionately high rates of stillbirths and childhood deaths at sites across seven countries in South Asia and sub-Saharan Africa (i.e., Baliakandi and Faridpur, Bangladesh; Kersa, Haramaya, and Harar, Ethiopia; Kisumu and Siaya, Kenya; Bamako, Mali; Manhiça and Quelimane, Mozambique; Makeni and Bo, Sierra Leone; and Soweto, South Africa).^16^ At each site, CHAMPS staff perform mortality surveillance in both the community (i.e., at the household level) and within healthcare facilities (i.e., outpatient clinics, community hospitals, and referral hospitals).

### Population inclusion and exclusion criteria.

Stillbirths (weighing ≥1,000 grams or born at ≥28 weeks’ gestation by last menstrual period or ultrasound dating when available, although less commonly) and live-born babies aged 0 to 27 days at the time of death (neonatal deaths) who were enrolled in CHAMPS from June 15, 2016 to December 31, 2023 were included. For the present analysis, deaths among infants and young children aged 1 to 59 months enrolled in CHAMPS were excluded because delays in healthcare seeking and provision among these populations in CHAMPS have been described previously.^10^ CHAMPS enrolls stillbirths and neonatal deaths in two ways. The first instance is cases in which the family of the deceased provides written informed consent for the deceased to undergo full enrollment, which includes extensive extraction of clinical data, verbal autopsy, postmortem testing, and determination of cause of death by an expert panel.^17^^,^^18^ For cases in which families do not consent to postmortem testing, data from clinical records and verbal autopsies are still collected, but no complete cause of death determination is conducted. Because the aim for the present study was to understand delayed illness recognition by caregivers, transport to healthcare facilities, and delays in healthcare facilities, both types of CHAMPS-enrolled cases were included; requisite data for these analyses were available for both types. However, analyses of delays stratified by causes of death were limited to those cases that underwent complete postmortem testing.

### Data sources.

For each enrolled stillbirth or neonatal death, CHAMPS staff conducted verbal autopsies in accordance with the WHO Verbal Autopsy form.^19^ This form contains both discrete and open-ended questions about signs and symptoms for the deceased before death, as well as questions about antemortem healthcare-seeking behaviors. CHAMPS staff also inquire about ANC and maternal illnesses for mothers of stillbirths and neonatal deaths. Additionally, for each enrolled case in CHAMPS, staff conduct an extensive review of all available antemortem clinical records (i.e., clinic notes, hospital admission forms, progress notes, laboratory and radiology results, etc.) for the mother, stillbirth (if records are available), and deceased neonate. Data from clinical charts are recorded in CHAMPS in both discrete and narrative summary form, noting both the clinical care provided as well as any deficits. Narrative and discrete variables collected in CHAMPS were used to identify delays that occurred before death, in accordance with the “Three Delays in Healthcare” framework. CHAMPS sites use a common protocol and provide standardized training on both conducting the verbal autopsy interview and extracting clinical record data.

### Variables.

Before extracting any data, the authors developed a list of variables that represented each delay in the “Three Delays-in-Healthcare” framework after reviewing variables identified in previous studies on illness recognition in the home and the decision to seek care (Delay 1), delays in identifying and transportation to a healthcare facility (Delay 2), and delays in the provision of quality clinical care (Delay 3). These variables were selected on the basis of the results of previous studies on delayed care seeking and provision conducted in similar settings, as well as experience among the co-authors, many of whom are panelists or coordinators for cause of death ascertainment meetings for stillbirths and neonates who died (Supplemental Table 1).^6^^–^^9^ The authors then piloted the use of their list of potential delays by reading narrative fields and discrete variables of 100 randomly selected cases across the included sites. During that phase, they iteratively added delays related to ANC, maternal conditions, illness recognition in the home, reaching a healthcare facility, and the provision of clinical care that were not part of the initial list (Supplemental Table 1). Facility deaths are defined in CHAMPS as those that occur in a healthcare facility. Community deaths are considered those that occur outside formal healthcare settings.

### Data extraction.

Two reviewers with backgrounds and expertise in global public health and global child health (N. Ramakrishna and C. A. Rees) independently read through narrative data fields from verbal autopsies and clinical summaries to identify a randomly selected set of ∼20% (*n* = 864) of all cases to extract data on documented delays as discrete variables when the specific delay of interest was documented. These cases were then reviewed by both reviewers to ensure the reproducibility of the extracted data. In rare cases of disagreement between these two reviewers, specific cases were reviewed collectively, and disagreements were discussed until consensus was achieved.

Assessing the documentation of delays was based on explicit mentions of delays in the reviewed narratives. For instance, the reviewers counted “Inadequate clinical care for baby documented” only in circumstances in which explicit mention was made of lacking clinical care (e.g., “there was no supplemental oxygen for the baby when they were struggling to breathe”). Specific delays that fell under each variable were collapsed for ease of analysis (e.g., “there was no supplemental oxygen for the baby when they were struggling to breathe” and “the needed medication was not available for my baby”). In cases in which there was no explicit mention of the candidate variables related to delays, no delays were counted for the respective case. In cases in which one reviewer had a question about the potential inclusion of a delay, both reviewers met to review specific cases together to discuss and reach consensus.

The authors then experimented with two state-of-the-art open-source large language models (LLAMA-3-70B and Mixtral 8 × 7B)^20^^,^^21^ to assess their ability to extract documented delays in the verbal autopsy and clinical narrative fields compared with the results of the independent, two-reviewer, manual review as a reference standard. They selected the large language model (LLAMA-3-70B) that exhibited a mean precision of >0.95 and a mean F_1_ score of >0.95 on 50 cases compared with the manual review (Supplemental Table 2). The unit of analysis for assessing model performance was each extracted variable in the included cases. During the training phase, the model was run on 20% of cases reviewed by two reviewers, and any discrepancies between the model’s output and the manual review were resolved through consensus among the reviewers. Then, the model prompt was fine-tuned on the basis of patterns observed in discrepancies caused by model errors (i.e., adjustments to the questions posed to LLAMA-3-70B based on observations of potential misinterpretations by the model; Supplemental Table 3). This prompt-optimized LLAMA-3-70B model was then used to extract data on identified delays from the remaining 80% of cases.

A subset of the remaining 80% of cases that were not reviewed by the two reviewers was initially reviewed again by the expert reviewers (N. Ramakrishna and C. A. Rees) to identify any additional discrepancies between the model’s output and manual review. This review was conducted on 50 cases across 101 extraction questions, representing 5,050 reviewed case–question outputs and resulting in a precision of >0.95 and an F_1_ score >0.95 (per-variable results are reported in Supplemental Table 2). Thus, data generated from the LLAMA-3-70B model were used for the study analyses.

#### Analyses.

Descriptive statistics were used to describe the demographics of enrolled cases and determine the frequency of each delay type using the “Three Delays in Healthcare” framework. On the basis of previous evidence suggesting that delays in healthcare-seeking may vary by the sex of a child and by site, delay frequencies were compared using these factors.^10^^,^^22^^–^^24^ The frequency of each class of delay was also plotted by year to assess trends over time. Furthermore, the authors assessed whether delays within the “Three Delays in Healthcare” framework were more common among mothers with certain maternal conditions (e.g., hypertension, etc.) and, post hoc, whether gestational age or birth weight was available. Analyses by cause of death were based on the 20 most common causes of death present anywhere in the causal chain of death (i.e., immediate, underlying, or comorbid) as determined via expert panel review of all postmortem data. χ^2^ tests were used to determine if delays varied by CHAMPS site, sex, and location of death (i.e., community or facility). When multiple related comparisons were presented, *P*-values were adjusted using the Benjamini-Hochberg false discovery rate method within each table. Stillbirths and neonatal deaths were analyzed separately because they were thought to be due to different types of delays. All analyses were conducted using R version 4.1.2 (R Foundation; Vienna, Austria), and two-sided adjusted *P* <0.05 was considered statistically significant.

## RESULTS

CHAMPS enrolled 11,633 total cases during the study period; 3,231 were infants and children and were excluded, and 1,323 cases lacked sufficient information to determine if delays occurred before stillbirth or neonatal death (Figure 1). Thus, 7,079 cases were included in the study analyses (stillbirths: *n* = 3,424; neonatal deaths: *n* = 3,655). The 7,079 included cases did not differ from the 1,323 cases with insufficient information to determine delays by sex (*P* = 0.13); however, there were more community deaths among excluded cases (49.3%) than among cases with sufficient information on delays to be included in the analyses (15.5%; *P* <0.001).

**Figure 1.**
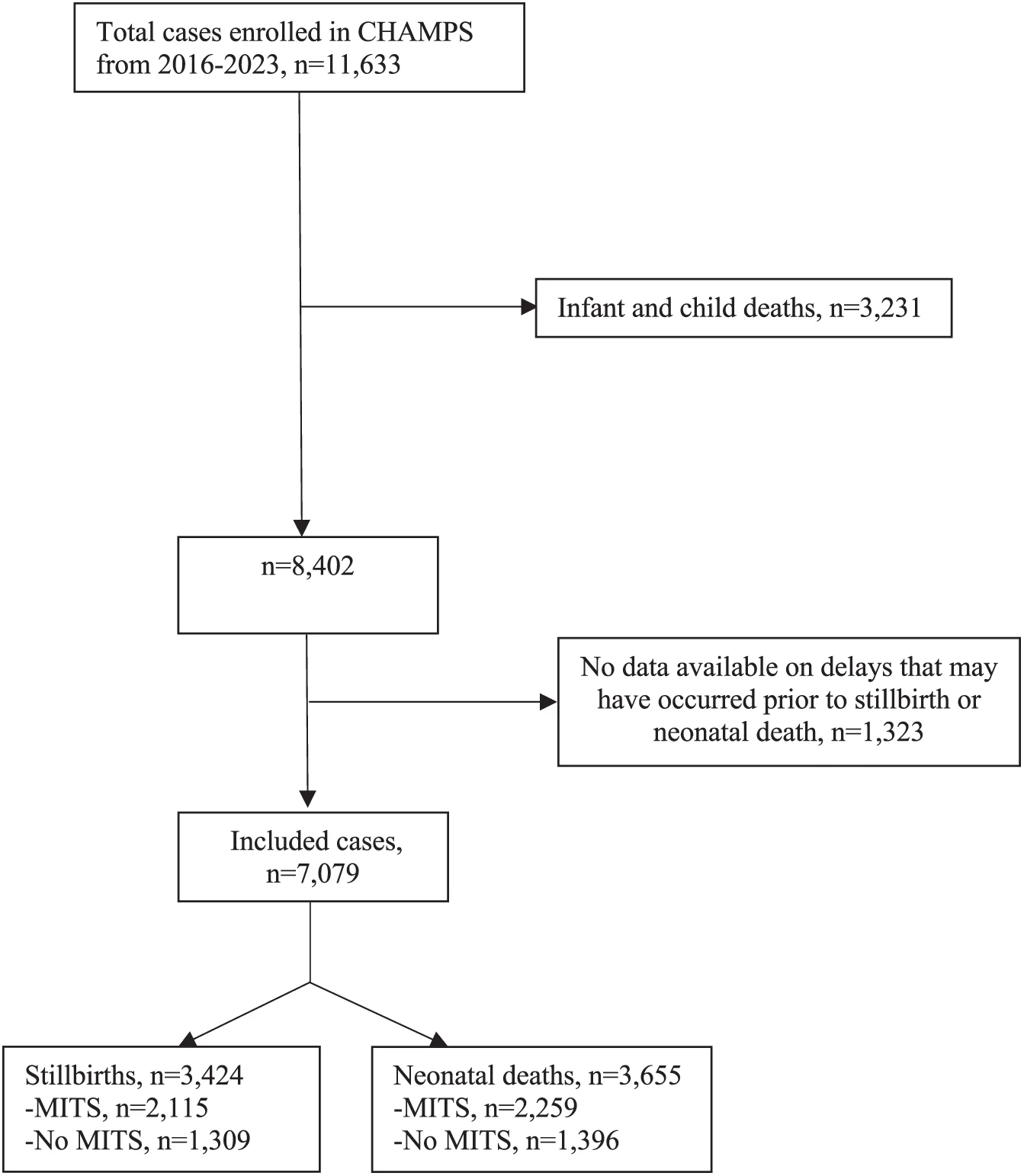
Preferred Reporting Items for Systematic Reviews and Meta-Analyses flow diagram for stillbirths and neonatal deaths included in analyses of the “Three Delays in Healthcare” framework in the CHAMPS network, 2016–2023.

Among included stillbirths, 53.6% were male, the median gestational age was 36 weeks (interquartile range [IQR]: 32–38 weeks), the median birth weight was 2,300 grams (IQR: 1,520–3,000 grams), and 84.9% of these stillbirths occurred in healthcare facilities (Table 1). Of the 3,424 stillbirths included in the analysis, stillbirth type was available for 2,037 cases (59.5%); among these, 1,085 (53.3%) were classified as fresh stillbirths, and 952 (46.7%) were classified as macerated.

**Table 1:** Description of stillbirths and neonatal deaths enrolled in the CHAMPS network

Characteristic	Stillbirths (*N* = 3,424), *n* (%)	Neonatal Deaths (*N* = 3,655), *n* (%)
Documented maternal gestational age (weeks; by ultrasound or last menstrual period)		
<28	211 (6.2)	462 (12.6)
28–33	497 (14.5)	470 (12.9)
34–36	482 (14.1)	356 (9.7)
37–42	1,026 (30.0)	1,027 (28.1)
Unknown	1,208 (35.3)	1,340 (36.7)
Median, interquartile range (IQR)	36 (32–39)	36 (30–38)
Age at death		
First 24 hours	–	1,459 (39.9)
1–6 days	–	1,494 (40.9)
7–27 days	–	702 (19.2)
Median, IQR	–	2 (0–5)
Sex		
Male	1,836 (53.6)	2,095 (57.3)
Female	1,564 (45.7)	1,551 (42.4)
Indeterminate or ambiguous	5 (0.1)	4 (0.1)
Unknown	19 (0.6)	5 (0.1)
HIV exposure status in stillbirth or neonate*		
Infected	6 (0.2)	11 (0.3)
Exposed and uninfected	191 (5.6)	403 (11.0)
Uninfected; exposure status negative/unknown	3,227 (94.2)	3,241 (88.7)
Delivery type		
Vaginal	2,464 (72.0)	2,470 (67.6)
C-section	684 (20.0)	854 (23.4)
Instrumentation (e.g., forceps)	1 (0.0)	2 (0.1)
Unknown	275 (8.0)	329 (9.0)
Birth weight (grams)		
Extremely low birth weight (<1,000)	145 (4.2)	382 (10.5)
Very low birth weight (1,000–1,499)	399 (11.7)	525 (14.4)
Low birth weight (1,500–2,499)	845 (24.7)	821 (22.5)
Normal weight (2,500–4,000)	1,127 (32.9)	1,359 (37.2)
Macrosomia (>4,000)	84 (2.5)	41 (1.1)
Unknown	824 (24.1)	527 (14.4)
Median, IQR (grams)	2,335 (1,569–3,000)	2,200 (1,300–3,000)
Location of birth		
Healthcare facility	2,906 (84.9)	3,029 (82.9)
Home	316 (9.2)	432 (11.8)
On the way to a health center	25 (0.7)	28 (0.8)
Other location	16 (0.5)	7 (0.2)
Unknown	161 (4.7)	159 (4.4)
CHAMPS site		
Bangladesh	850 (24.8)	807 (22.1)
Ethiopia	639 (18.7)	479 (13.1)
Kenya	290 (8.5)	315 (8.6)
Mali	476 (13.9)	447 (12.2)
Mozambique	537 (15.7)	684 (18.7)
Sierra Leone	326 (9.5)	343 (9.4)
South Africa	306 (8.9)	580 (15.9)
MITS procedure performed and causes of death determined	2,115 (61.8)	2,259 (61.8)
Site of death		
Healthcare facility	3,009 (87.9)	2,975 (81.4)
Community	415 (12.1)	680 (18.6)
Hospital duration (hours), median (IQR), *N* = 2,778	–	28 (8–79)
Weight at MITS (kilograms), median (IQR), *N* = 4,343	2.1 (1.4–2.8)	1.9 (1.2–2.8)

CHAMPS = Child Health and Mortality Prevention Surveillance; IQR = interquartile range; MITS = minimally invasive tissue sampling.

* HIV infection/exposure status refers to the stillbirth or neonate. HIV infection was defined via postmortem HIV DNA and/or RNA polymerase chain reaction testing of blood. HIV-exposed and uninfected indicates documented HIV exposure with no HIV infection detected in the stillbirth or neonate. The “uninfected; exposure status negative/unknown” category includes cases with no HIV infection detected for whom HIV exposure was either not documented or documented as absent.

Among neonatal deaths, 57.3% were male, the median gestational age at birth was 34 weeks (IQR: 30–38 weeks), and the median birth weight was 2,060 grams (IQR: 1,235–2,950 grams; Table 1). The majority (82.9%) of neonatal deaths were among neonates who were born in healthcare facilities, and 81.4% of the deaths occurred in healthcare facilities. The median age at the time of death among neonatal deaths was 2 days (IQR: 0–5 days).

### Overall delays in the “Three Delays in Healthcare” framework.

Documented inadequate clinical care (e.g., delays in neonatal resuscitation, infrastructure limitations leading to suboptimal clinical care, etc.) was the most commonly identified delay for stillbirths (Figure 2). Family underestimation of the neonate’s illness severity was the most common delay among neonatal deaths. Overall, 96.8% (*n* = 3,313) of stillbirths and 97.7% (*n* = 3,571) of neonatal deaths had ≥1 delay identified (Supplemental Tables 4 and 5). A total of 240 (7.0%) stillbirths and 1,389 (38.0%) neonatal deaths had ≥1 delay identified in all three categories in the “Three Delays in Healthcare” framework.

**Figure 2.**
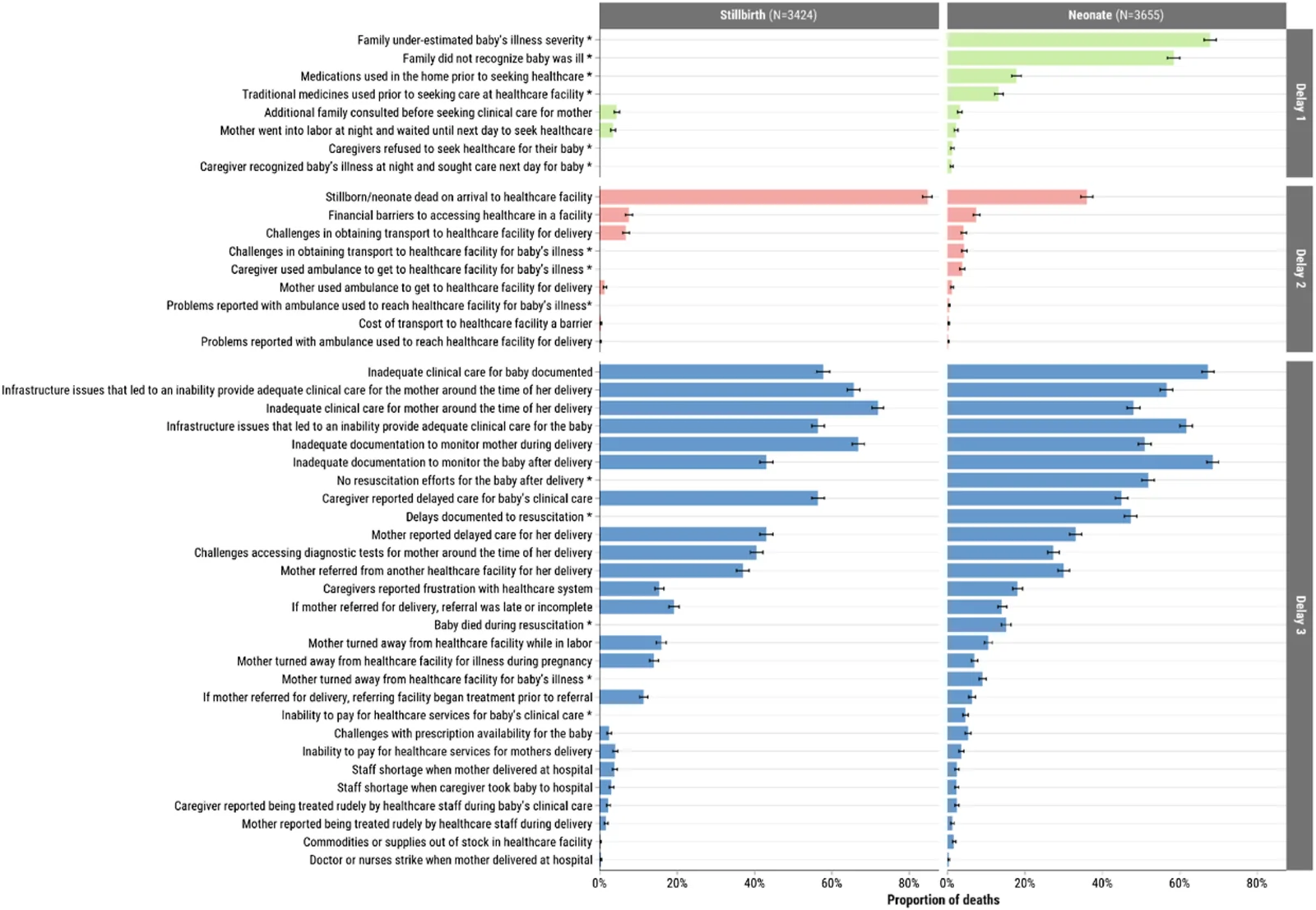
Proportion of included stillbirths and neonatal deaths that experienced each type of delay in the “Three Delays-in-Healthcare” framework.

The most common delays in neonatal deaths varied by the time of death (Supplemental Figure 1). For example, delays in the home (e.g., underestimation of illness risk) were more common among neonates who died at age 7 to 27 days, and delays in quality clinical care (e.g., inadequate monitoring of mother during delivery and of baby after delivery) were more common among neonates who died within the first 24 hours of life. The proportion of cases with at least one delay did not vary substantially by year (Supplemental Figure 2).

Delays were not more common among male stillbirths compared with female stillbirths (Supplemental Table 4) or among male neonatal deaths compared with female neonatal deaths (Supplemental Table 5). Delays in illness recognition and in reaching a healthcare facility were more common among stillbirths and neonatal deaths that occurred in the community than among those that occurred in healthcare facilities (Supplemental Tables 4 and 5).

The frequency of delays varied by availability of either gestational age or birth weight data (Supplemental Table 6). Among stillbirths, Delays 1 (49.3% versus 55.0%; *P* = 0.029) and 2 (85.5% versus 91.0%; *P* = 0.002) were more frequent when both gestational age and birth weight were unavailable, whereas Delay 3 and any delay were similar. Among neonatal deaths, Delay 2 (42.0% versus 54.1%; *P* <0.001), Delay 3 (95.0% versus 98.0%; *P* = 0.011), and any delay (97.5% versus 99.4%; *P* = 0.023) were more frequent when both measures were unavailable; Delay 1 did not differ significantly.

### Site variation in delays in the “Three Delays in Healthcare” framework.

The proportion of stillbirths and neonates who experienced delays before death varied by site (Figure 3). Overall, delays in illness recognition, reaching a healthcare facility, and the provision of clinical care were most common among stillbirths and neonatal deaths that were enrolled in Bangladesh (*P* <0.001 compared with other sites; Supplemental Tables 4 and 5). Delays in the provision of healthcare were least common for stillbirths and neonatal deaths in South Africa (Supplemental Figure 3). There was no substantial difference in delays by sex of the deceased and site (Figure 3).

**Figure 3.**
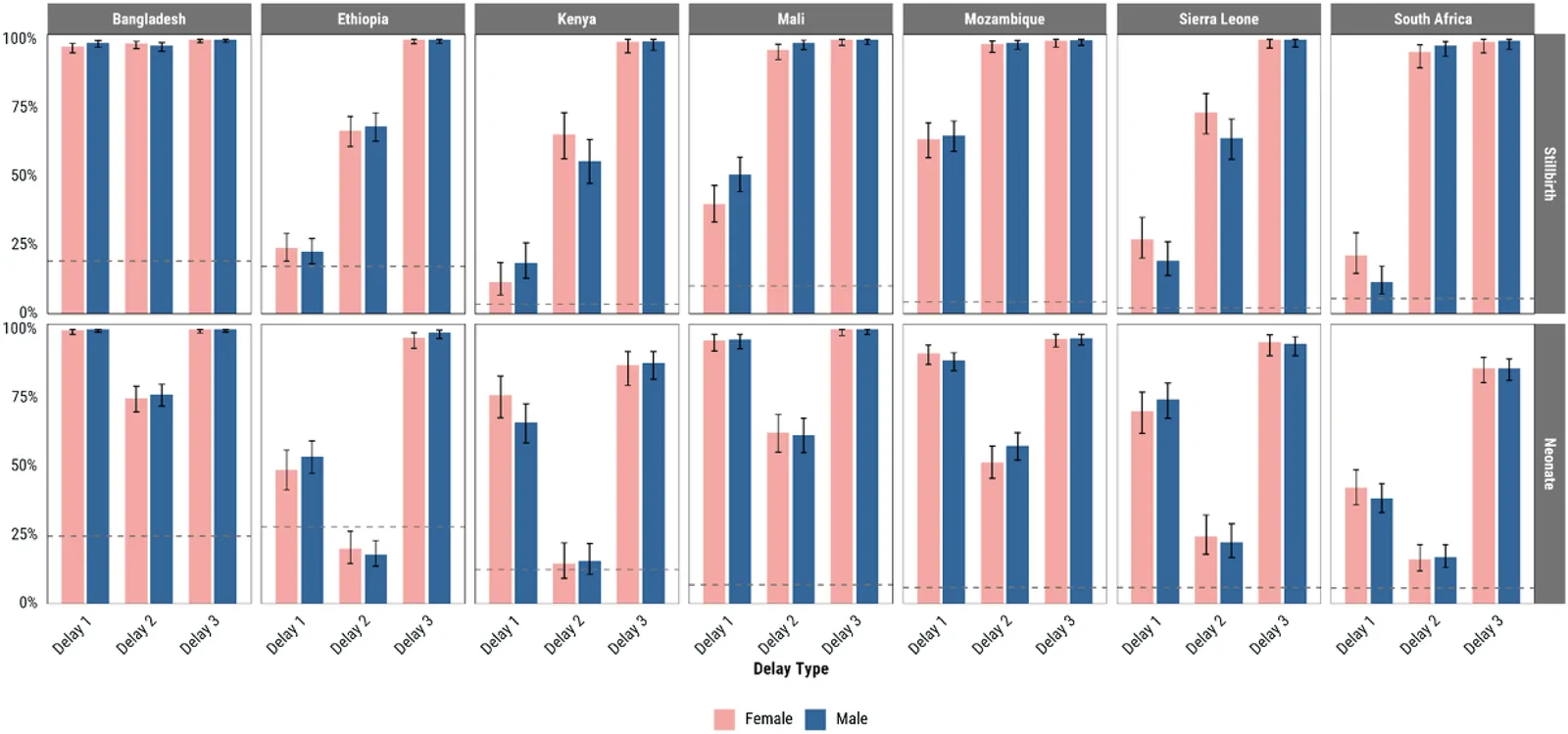
Proportion of stillbirths and neonates who died and experienced each delay in the “Three Delays-in-Healthcare” framework by site and sex. * Bars represent the proportion of deaths with at least one delay identified within each category. Error bars indicate 95% CIs. Dashed horizontal lines indicate the proportion of deaths that occurred after home deliveries in each site and age group.

### Variation in delays by cause of death for stillbirths and neonatal deaths.

Among the 2,115 stillbirths who underwent the full postmortem determination of cause of death procedures, the most common causes of stillbirth anywhere in the causal chain included intrauterine asphyxia/hypoxia (*n* = 1,696; 80.2%), congenital birth defects (*n* = 184; 8.7%), and sepsis (*n* = 109; 5.2%). Among stillbirths, the most commonly identified maternal conditions included maternal hypertension (*n* = 2,718; 79.4%), placental complications (*n* = 2,094; 61.2%), and anemia (*n* = 1,570; 45.9%).

Among the 2,259 neonates who underwent the full postmortem determination of cause of death procedures, the most common causes of death anywhere in the causal chain included preterm birth complications (*n* = 933; 44.1%), perinatal asphyxia/hypoxia (*n* = 859; 40.6%), and sepsis (*n* = 798; 37.7%). The most common maternal conditions in neonatal deaths included hypertension (*n* = 1,901; 52.0%), multiple gestation (*n* = 1,370; 37.5%), and placental complications (*n* = 1,168; 32.0%). Types of delays did not differ substantially by maternal condition (Figure 4). There was no substantial variation in the specific delays experienced by stillbirths (Supplemental Figure 4) or neonates (Supplemental Figure 5) when grouped by causes of death.

**Figure 4.**
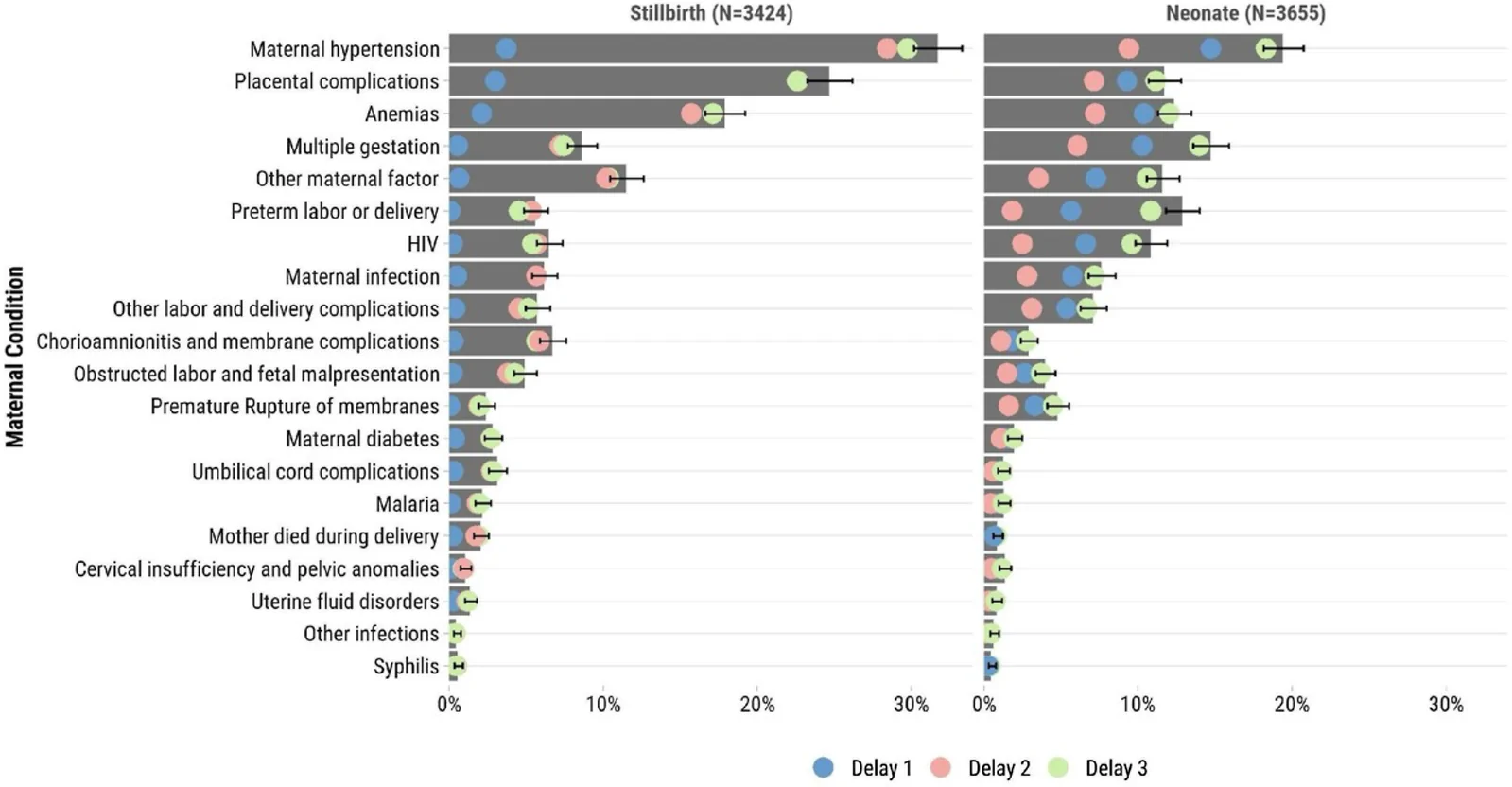
Maternal conditions and delays among 1) stillbirths and 2) neonatal deaths. * Bars indicate the proportions of stillbirths/neonates with each maternal condition, and dots indicate the proportions of each delay relative to the bar length.

### Antenatal care for stillbirths and neonatal deaths.

Among the 3,424 stillbirths, 73.7% of mothers had ≥1 ANC visit documented, and only 66.3% of the 3,655 mothers whose neonate died had ≥1 ANC visit documented (Figure 5). Nearly half of mothers of stillbirths (48%) and 41% of mothers of deceased neonates had only one to three ANC visits. Only 54 (1.6%) stillbirths and 33 (0.9%) neonatal deaths had documentation of eight or more ANC visits, as recommended by the WHO.^25^ The median number of ANC visits was two (IQR: 1–4 visits) for both stillbirths and neonatal deaths. Antenatal care visits were least common during the first trimester and most common during the third trimester of pregnancy for both stillbirths and neonatal deaths. The proportion of included cases with any documented ANC visits was lowest among cases enrolled at the Ethiopia site and highest among cases enrolled at the Bangladesh site (Supplemental Figure 6).

**Figure 5.**
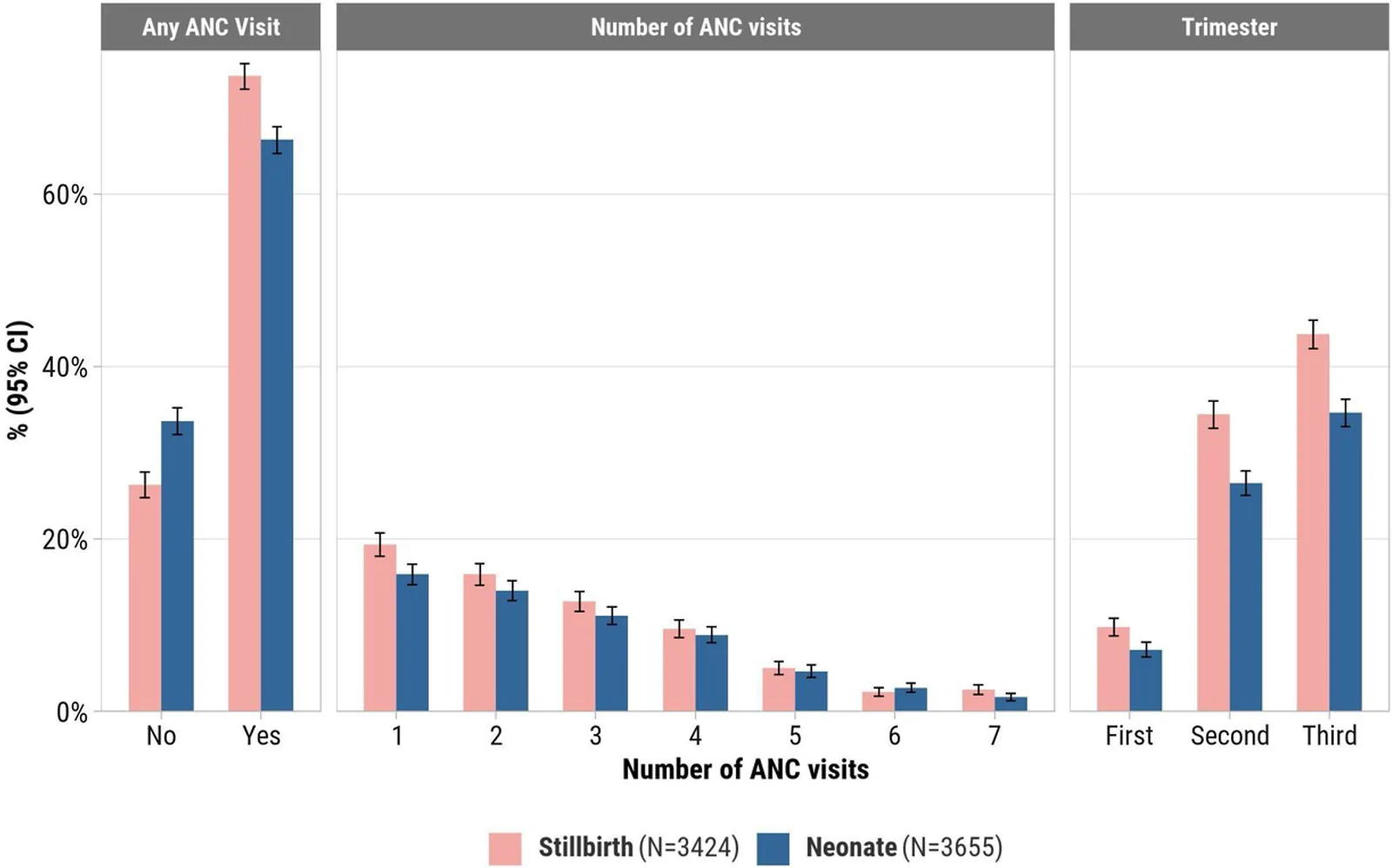
Distribution and timing of antenatal care visits for mothers of stillbirths (*N* = 3,424) and neonates who died (*N* = 3,655). CHAMPS = Child Health and Mortality Prevention Surveillance; DeCoDe = Determination of Cause of Death; IQR = interquartile range; LMICs = low- and middle-income countries; MITS = minimally invasive tissue sampling.

## DISCUSSION

In the present study of 3,424 stillbirths and 3,655 neonatal deaths in regions with high rates of childhood mortality in seven low- and middle-income countries, delays in the decision to seek care, delays in reaching healthcare facilities, and delays in the provision of healthcare before death occurred in almost all cases. Delays occurring in healthcare facilities were most common. Although all three types of delays were common across the CHAMPS network, specific types of delays varied by site. Surprisingly, delay frequency did not change substantially during the study period, which included the coronavirus disease 2019 pandemic, a time of substantial health system disruption globally and in many of the study settings.^26^^,^^27^ Contrary to results from previous studies, differences in delays by the sex of the stillbirth or neonatal death were not observed.^10^^,^^22^^–^^24^ This may have been because previous studies on differences in sex have included older children and the present study was restricted to stillbirths and neonatal deaths, populations in which sex-based differences may be less pronounced. The WHO encourages eight ANC visits during pregnancy because there is evidence associating frequent ANC visits with reduced perinatal deaths and improved maternal outcomes; however, ANC among mothers of stillbirths and neonatal deaths was infrequent and often began late in pregnancy.^25^

Previous studies conducted in the CHAMPS network have revealed that the local expert review panels that review each death frequently note that addressing delayed access to, and suboptimal, clinical care is the most common way in which stillbirths and neonatal deaths could have been averted.^28^ Consistent with previous studies conducted in Kenya,^29^ delayed provision of high-quality clinical care was the most common type of delay experienced by stillbirths and neonatal deaths. Previous modeling studies have indicated that health service improvements may have the greatest impact on reducing neonatal mortality in low- and middle-income countries.^30^ Furthermore, results from a previous systematic review suggest that common delays related to maternal and newborn care in healthcare facilities, including a lack of appropriate storage for medications, a lack of equipment, and limited surgical, transfusion, and laboratory facilities, are common delays in healthcare facilities in sub-Saharan Africa and South Asia.^31^ Thus, additional efforts to improve health services available to pregnant mothers during pregnancy to enhance the detection of maternal conditions around the time of delivery and to neonates are warranted.^32^

Although site-level variation in clinical documentation and potential differences in the content of verbal autopsy narratives may explain some of the observed site-level variation in delays, the study findings also suggest that a site-specific tailored approach to health services strengthening may be indicated. Resources needed to monitor mothers during delivery and equipment for neonatal resuscitation vary in sub-Saharan Africa and Bangladesh and even within countries. Thus, an assessment of needed resources should be conducted at the site level. The observed site variation suggests that interventions tailored to illness recognition for mothers may be better suited for some CHAMPS sites (e.g., Bangladesh, Mali, and Mozambique), whereas improved healthcare provision may be most needed at other CHAMPS sites (e.g., Sierra Leone and Bangladesh). A greater frequency of delays was also observed among stillbirths and neonates with missing gestational age or birth weight data, which may be a marker of more suboptimal clinical care among cases with worse clinical documentation. Although the present study was not designed to elucidate reasons for delayed healthcare provision among stillbirths and neonatal deaths, the authors of future assessments may focus on structural and systems-level characteristics of each country (and more specifically each region) to design targeted interventions to prevent future delays.

In the present study, <10% of mothers of stillbirths and neonates who died had their first ANC visit during the first 12 weeks of pregnancy, and very few had the recommended eight ANC visits. Because frequent ANC visits may enhance the timely detection and management of potential pregnancy-related complications, improve communication between healthcare providers and pregnant women, and increase the likelihood of positive pregnancy outcomes, the WHO recommends that the first ANC visit occur before the 12th week of pregnancy and that expecting mothers have at least eight ANC visits during pregnancy.^25^ The present study’s findings of infrequent ANC visits among mothers of stillbirths and neonates who died align with findings from previous studies from Kenya and Bangladesh that suggest that 50% to 60% of mothers of neonates who survive have four or more ANC visits during their pregnancy.^33^^,^^34^ Thus, the frequency of ANC visits in the present study cannot be interpreted as a risk factor for stillbirth or neonatal deaths in the absence of a comparison to surviving births. Meta-analysis-level data suggest that ANC visits in sub-Saharan Africa reduce the risk of neonatal mortality by as much as 39%.^35^ Moreover, results from a study in Ethiopia suggest that high-quality ANC may reduce the odds of a mother having a stillbirth by as much as 80%.^36^ Thus, additional efforts may be needed to augment both the frequency and quality of ANC visits in settings like those in which CHAMPS operates. Such efforts may include broadly promoting the benefits of ANC, integrating ANC with clinical care for chronic diseases, or linking pregnancy care with community health workers, as demonstrated in previous studies.^37^

### Limitations.

The results of the present multi-country study should be interpreted in light of certain limitations. Given the observational nature of the study, no causality can be inferred between the observed delays and stillbirth or neonatal death. The authors were unable to compare the frequency of delays among cases of stillbirths or neonatal deaths to live births or to neonates who survived because CHAMPS only enrolls mortality cases. Thus, it is unclear whether the identified delays are more common among mortality cases than among survivors. It is possible that some observed site-level variation in delays may reflect differences in clinical documentation and what is shared in verbal autopsies, despite CHAMPS using a standardized protocol across sites. Moreover, delays were not compared between fresh and macerated stillbirths, which may be considered in future analyses. There were more community deaths than facility deaths excluded from the analyses because of missing data needed to determine the frequency of delays in this population. Furthermore, it is possible that there were additional, unmeasured delays among >7,000 stillbirths and neonatal deaths, particularly because the data sources included narrative reviews to capture delays. Thus, the estimates of delays in healthcare seeking and provision may be underestimates of such occurrences. Given the outcome of all included cases (i.e., death), the review process may also have been subject to confirmation bias. The authors mitigated this risk by applying strict requirements for documenting a delay. They also could not assess the potential role of seasonality in documented delays.

The frequency of ANC visits among mothers of stillbirths and neonates who died could not be compared with the frequency of such visits among neonates who survived. Illness recognition and decisions to seek healthcare are often influenced by culture and societal norms,^38^^,^^39^ which could not be measured in the present study. In many cases, the specific elements of clinical care that were delayed were not recorded. Thus, additional studies are warranted to understand specific delays in clinical care administration. The authors could not determine if maternal diagnoses were missed during ANC visits and the potential contribution of such missed diagnoses to stillbirths or neonatal deaths. A rigorously validated large language model was used to extract discrete variables from narrative fields, which may have introduced some assumptions made by the model. The authors addressed this issue by iteratively training the model on the study questions and manually reviewing a large proportion of cases to ensure that the model did not hallucinate or make inappropriate inferences regarding delays experienced before the stillbirth or death of the neonate.

## CONCLUSION

Delayed illness recognition and healthcare-seeking by caregivers and delayed healthcare provision were common among stillbirths and neonatal deaths in seven low- and middle-income countries. Additional efforts are warranted to promote early and complete ANC among mothers. Despite high facility delivery rates, delays in healthcare provision were most predominant, suggesting that improving the quality of facility-based clinical care, rather than simply increasing facility access, may be the most impactful intervention for reducing perinatal mortality in these settings.

## Supplemental Materials

10.4269/ajtmh.26-0164 Supplemental Materials
