# Supplementary material for: Delays in Illness Recognition, Healthcare Seeking, and Healthcare Provision for Stillbirths and Neonatal Deaths in Seven Low- and Middle-Income Countries
Source: Am J Trop Med Hyg. 2026 Oct 22:tpmd260164. Online ahead of print. doi: 10.4269/ajtmh.26-0164 (PMC13694915; doi:10.4269/ajtmh.26-0164)

Supplemental Table 1. Candidate delays in the "Three Delays-in-Healthcare" framework identified through review of prior studies and through pilot review of cases of stillbirths and neonatal deaths

	Variables Identified through Review of Prior Literature[6–9]	Variables Identified through Pilot Review of Cases
**Delay 1**<br>Illness recognition in the home/decision to seek care	-Family recognized baby was severely ill (e.g., "my baby was very sick")<br>-Family under-estimation of illness severity (e.g., "I did not know my baby was so sick that they could die")<br>-Caregivers refused to seek healthcare for their baby (e.g., "we had already seen a doctor for this illness so we did not want to go back")<br>-Medications used in the home prior to seeking care<br>-Traditional medicines used prior to seeking care at healthcare facility<br>-Additional family members consulted before seeking care for mother (e.g., "I needed approval of my mother/grandmother to go to the clinic")	-Mother went into labor at night and waited until next day to seek healthcare<br>-Caregiver recognized baby's illness at night and sought care next day for baby
**Delay 2**<br>Identifying and transportation to a healthcare facility)	-Financial barriers to accessing healthcare in a facility<br>-Cost of transport to healthcare facility a barrier<br>-Challenges in obtaining transport to healthcare facility for delivery<br>- Challenges in obtaining transport to healthcare facility for baby's illness<br>-Long distance to healthcare facility*<br>-Competing obligations that prevented transport to healthcare facility*<br>-Family turned away from healthcare facility for delivery*<br>-Family turned away from healthcare facility for baby's illness*<br>-Concern for personal safety in transport*	-Stillborn/neonate dead on arrival to healthcare facility<br>-Delay in caregiver use of ambulance to get to healthcare facility for delivery<br>-Delay in caregiver use of ambulance to get to healthcare facility for baby's illness<br>-Problems reported with ambulance used to reach healthcare facility for baby's illness
**Delay 3**<br>The provision of quality clinical care	-Inadequate clinical care for baby documented (e.g., "there was no supplemental oxygen available for the baby")<br>-Infrastructure issues that led to an inability to provide adequate clinical care for the mother around the time of her delivery (e.g., "the electricity was not functional at the time").<br>-Inadequate clinical care for mother around the time of her delivery (e.g., "there was no healthcare provider present at the time of delivery")<br>-Infrastructure issues that led to an inability to provide adequate clinical care for the baby (e.g., "the electricity was not functional at the time").<br>-Inadequate documentation to monitor mother during delivery (e.g., "there was no partograph available")<br>-Inadequate documentation to monitor baby after delivery (e.g., "there were no warmers or monitors available for the baby")<br>-Caregiver reported delayed care for baby's clinical care (e.g., "they called for the doctor but they did not come")<br>-Challenges accessing diagnostic tests for mother around the time of delivery (e.g., "there were no lab test results available")<br>-Baby died during resuscitation<br>-Inability to pay for healthcare services for baby's clinical care<br>-Inability to pay for healthcare services for mother's delivery<br>-Challenges with prescription availability for the baby<br>-Staff shortage when mother delivered at hospital<br>-Staff shortage when caregiver took baby to hospital<br>-Commodities or supplies out of stock in healthcare facility (e.g., "they told me there was a stockout of the medicines")	-No resuscitation efforts for the baby after delivery<br>-Mother referred from another healthcare facility for her delivery<br>-If mother referred for delivery, referral was late or incomplete (e.g., "we were referred but the other hospital did not know about us")<br>-If mother referred for delivery, referring facility began treatment prior to referral<br>-Mother turned away from healthcare facility while in labor<br>-Mother turned away from healthcare facility for illness during pregnancy<br>-Mother turned away from healthcare facility for baby's illness<br>-Caregivers reported frustration with healthcare system (e.g., "we were frustrated with the delays in being seen in the hospital")<br>-Caregiver reported being treated rudely by healthcare staff during baby's clinical care (e.g., "I felt ignored and treated poorly when asking for help for my baby")<br>-Doctor or nurses strike when mother delivered at hospital

*assessed based on prior literature but not documented in reviewed cases

Supplemental Table 2. Performance of LLAMA-3-70B validation results in comparison to manual review of 50 cases

Type	Variable	Positive, n	Negative, n	Precision	Recall	F1
Binary	Was the mother sick during pregnancy?	11	39	0.846	1.000	0.917
Binary	Was the mother 35 years old or older during the pregnancy?	1	49	0.500	1.000	0.667
Binary	Did the mother have gestational diabetes?	0	50	N/A	N/A	N/A
Binary	Did the mother have anemia during pregnancy?	3	47	0.500	1.000	0.667
Binary	Did the mother have covid-19 during her pregnancy?	0	50	N/A	N/A	N/A
Binary	Did the mother have fibroids during pregnancy?	0	50	N/A	N/A	N/A
Binary	Did the mother have a sexually transmitted infection (sti) during pregnancy?	2	48	1.000	1.000	1.000
Binary	Did the mother have malaria during this pregnancy?	0	50	N/A	N/A	N/A
Binary	Did the mother have hiv during her pregnancy?	2	48	0.667	1.000	0.800
Binary	Did the mother have abruprio placenta during pregnancy?	1	49	1.000	1.000	1.000
Binary	Was this a twin pregnancy?	2	48	1.000	1.000	1.000
Binary	Did the mother have triplets during this pregnancy?	0	50	N/A	N/A	N/A
Binary	Did mother have high blood pressure or hypertension during pregnancy?	1	49	1.000	1.000	1.000
Binary	Did mother have pre-eclampsia, eclampsia, pet, or hellp during pregnancy?	1	49	0.500	1.000	0.667
Binary	Did the mother see a doctor or have a clinic visit specifically for her own illness during pregnancy (not during labor)?	13	37	0.722	1.000	0.839
Generic	If the mother saw a doctor or had a clinic visit for her illness during pregnancy, were any deficits in the quality of clinical care during those visits documented?	N/A	N/A	N/A	N/A	N/A
Generic	What were the deficits in the quality of clinical care during the doctor or the clinic visit for her illness during pregnancy?	N/A	N/A	N/A	N/A	N/A
Binary	Did mother have any anc visits?	20	30	1.000	1.000	1.000
Quantitative	How many anc visits were documented?	N/A	N/A	N/A	N/A	N/A
Binary	Did the mother have an antenatal care (anc) visit in the first trimester of this pregnancy?	1	49	1.000	1.000	1.000
Binary	Did the mother have an antenatal care (anc) visit in the second trimester of this pregnancy?	12	38	1.000	1.000	1.000
Binary	Did the mother have an antenatal care (anc) visit in the third trimester of this pregnancy?	17	33	1.000	1.000	1.000
Quantitative	How far along in the pregnancy was the mother when she learned that she was pregnant?	N/A	N/A	N/A	N/A	N/A
Binary	Did the mother deliver this baby at home?	7	43	1.000	1.000	1.000
Binary	Did the mother deliver this baby at a healthcare facility?	36	14	1.000	1.000	1.000
Binary	Did the mother die during the delivery of this baby?	0	50	N/A	N/A	N/A
Binary	Was the birth attended by a healthcare provider?	36	14	1.000	1.000	1.000
Generic	What kind of healthcare provider attended the delivery of this baby?	N/A	N/A	N/A	N/A	N/A
Binary	Was the mother sent home from a healthcare facility while she was in labor?	1	49	1.000	1.000	1.000
Binary	Did the mother delivery by c-section?	7	43	1.000	1.000	1.000
Binary	Did the mother delivery by vaginal delivery?	18	32	1.000	1.000	1.000
Binary	Did the family recognize their baby was ill?	12	38	1.000	1.000	1.000
Binary	Did the family underestimate the severity of the baby's illness?	11	39	0.917	1.000	0.957
Binary	Did family use traditional medicine prior to seeking care at healthcare facility for the baby?	2	48	1.000	1.000	1.000
Binary	Did the caregivers give the baby any medications prior to taking their baby to a clinic or healthcare facility?	2	48	1.000	1.000	1.000
Binary	Did the family have any financial difficulties that made it challenging to access healthcare?	6	44	1.000	1.000	1.000
Quantitative	After recognizing that the baby was sick, how much time passsed before they sought healthcare?	N/A	N/A	N/A	N/A	N/A
Binary	Did additional family need to be consulted before mother could seek care for herself?	1	49	1.000	1.000	1.000
Binary	Did additional family need to be consulted before mother could seek care for the baby?	1	49	1.000	1.000	1.000
Binary	Did the caregivers express frustrations with the healthcare system?	0	50	N/A	N/A	N/A
Generic	If the caregiver expressed frustrations with the healthcare system, what were those frustrations?	N/A	N/A	N/A	N/A	N/A
Binary	Did the caregivers refuse to seek care for their baby?	0	50	N/A	N/A	N/A
Generic	Were there any other challenges in recognizing the illness	N/A	N/A	N/A	N/A	N/A
Binary	Did the caregivers report any challenges in obtaining transport to a healthcare facility for the delivery?	1	49	1.000	1.000	1.000
Generic	If the caregivers reported challenges in obtaining transport to a healthcare facility for the baby's delivery, what were those challenges?	N/A	N/A	N/A	N/A	N/A
Binary	Did the caregivers report any challenges obtaining transport to a healthcare facility for the baby's illness?	1	49	1.000	1.000	1.000
Generic	If the caregivers reported challenges obtaining transport to a healthcare facility for the baby's illness, what were those challenges?	N/A	N/A	N/A	N/A	N/A
Binary	Did the caregivers report any competing obligations that made it challenging to get to the hospital for the baby's delivery?	4	46	1.000	1.000	1.000
Generic	If the caregivers reported competing obligations that made it challenging to get to the hospital for the baby's delivery, what were those competing obligations?	N/A	N/A	N/A	N/A	N/A
Binary	Did the caregivers report any competing obligations that made it challenging to get to a healthcare facility for the baby's illness?	4	46	1.000	1.000	1.000
Generic	If the caregivers reported competing obligations that made it challenging to get to a healthcare facility for the baby's illness, what were those competing obligations?	N/A	N/A	N/A	N/A	N/A
Binary	Did the caregivers report that the cost of transport to a healthcare facility made it difficult to get to a healthcare facility?	1	49	1.000	1.000	1.000
Binary	Was the mother turned away from a healthcare facility while the mother was in labor?	0	50	N/A	N/A	N/A

Binary	Was the mother turned away from a healthcare facility for a visit for her own illness during pregnancy?	1	49	1.000	1.000	1.000
Binary	Was the caregiver turned away from a healthcare facility for a visit for the baby's illness?	0	50	N/A	N/A	N/A
Binary	Did the mother report that she waited to seek healthcare the next day while she was in labor because she went into labor at night?	0	50	N/A	N/A	N/A
Binary	Did the caregivers report that they waited to seek healthcare the next day because they recognized the baby's illness at night?	0	50	N/A	N/A	N/A
Binary	Did the caregivers report that they felt unsafe traveling to a healthcare facility at night?	0	50	N/A	N/A	N/A
Binary	Did the mother go in an ambulance to the hospital for the delivery of her baby?	0	50	N/A	N/A	N/A
Binary	Did the caregiver take an ambulance to a healthcare facility for their baby's illness?	0	50	N/A	N/A	N/A
Generic	If the mother took an ambulance for her delivery, were there any problems with the ambulance?	N/A	N/A	N/A	N/A	N/A
Generic	If the caregivers took an ambulance for their baby's illness, were there any problems with the ambulance?	N/A	N/A	N/A	N/A	N/A
Generic	Were there any other challenges in reaching a healthcare facility?	N/A	N/A	N/A	N/A	N/A
Binary	Did the caregivers seek clinical care at a healthcare facility for the baby's illness?	18	32	1.000	1.000	1.000
Binary	Was the stillborn/neonate dead when they arrived at a healthcare facility?	16	34	1.000	1.000	1.000
Binary	Was there reported inadequate documentation to monitor the mother during delivery?	16	34	0.889	1.000	0.941
Binary	Was there reported inadequate documentation to monitor the baby after delivery?	13	37	0.929	1.000	0.963
Binary	Was the mother referred from another healthcare facility for her delivery?	11	39	1.000	1.000	1.000
Generic	If the mother was referred from another healthcare facility for delivery, was the referral late or incomplete?	N/A	N/A	N/A	N/A	N/A
Generic	If the mother was referred from another healthcare facility for her delivery, did the referring facilty begin treatment prior to referral?	N/A	N/A	N/A	N/A	N/A
Binary	Was there any documentation of commodities or supplies being out of stock?	0	50	N/A	N/A	N/A
Generic	If commodities were out of stock, which commodities were out of stock?	N/A	N/A	N/A	N/A	N/A
Binary	Was an inability to pay for healthcare services for the mothers delivery documented?	4	46	1.000	1.000	1.000
Binary	Was an inability to pay for healthcare services for the baby's care documented?	3	47	1.000	1.000	1.000
Binary	Were any resuscitation efforts made for the baby after delivery?	17	33	1.000	1.000	1.000
Binary	Did the baby die during the initial resuscitation process?	0	50	N/A	N/A	N/A
Generic	Which resuscitative efforts were made for the baby before they died?	N/A	N/A	N/A	N/A	N/A
Binary	Were there any delays to the resuscitation for the baby?	8	42	1.000	1.000	1.000
Generic	What were the delays to the resuscitation for the baby?	N/A	N/A	N/A	N/A	N/A
Binary	Was there a doctor's or nurse's strike when the mother delivered at the hospital?	0	50	N/A	N/A	N/A
Binary	Was there a doctor's or nurse's strike when the caregiver took the baby to the hospital?	0	50	N/A	N/A	N/A
Binary	Was there a staff shortage when the mother delivered at the hospital?	1	49	1.000	1.000	1.000
Binary	Was there a staff shortage when the caregiver took the baby to the hospital?	0	50	N/A	N/A	N/A
Binary	Did the mother report that healthcare staff treated her rudely or inappropriately during delivery?	0	50	N/A	N/A	N/A
Binary	Did the caregiver report that healthcare staff treated them rudely or inappropriately when they took the baby to a healthcare facility?	0	50	N/A	N/A	N/A
Binary	Did the mother perceive that her clinical care was delayed in the hospital around the time of her delivery?	8	42	1.000	1.000	1.000
Binary	Did the caregivers perceive that the baby's clinical care was delayed?	9	41	1.000	1.000	1.000
Binary	Was there documentation of inadequate clinical care for the mother around the time of her delivery?	17	33	0.944	1.000	0.971
Binary	Was there documentation of inadequate clinical care for the baby?	17	33	1.000	1.000	1.000
Binary	Were there infrastructure issues that led to an inability provide adequate clinical care for the mother around the time of her delivery?	18	32	1.000	1.000	1.000
Binary	Were there infrastructure issues that led to an inability provide adequate clinical care for the baby?	16	34	1.000	1.000	1.000
Generic	If there were infrastructure issues that led to an inability provide adequate clinical care for the mother around the time of her delivery, what were they?	N/A	N/A	N/A	N/A	N/A
Binary	If there were infrastructure issues that led to an inability provide adequate clinical care for the baby, what were they?	0	50	0.000	N/A	N/A
Binary	Was there documentation of lack of adherence to clinical care guidelines for the mother's delivery?	0	50	0.000	N/A	N/A
Binary	Was there documentation of lack of adherence to clinical care guidelines for the baby's clinical care?	0	50	0.000	N/A	N/A
Binary	Were there problems accessing diagnostic tests for the mother around the time of her delivery?	4	46	1.000	1.000	1.000
Binary	Were there problems accessing diagnostic tests for the clinical care of the baby?	7	43	1.000	1.000	1.000
Binary	Were there problems with prescription availability for the baby?	0	50	N/A	N/A	N/A
Binary	Did the mother leave the healthcare facility against medical advice around the time of her delivery?	2	48	1.000	1.000	1.000
Binary	Did the caregiver take the baby home from the healthcare facility against medical advice?	0	50	N/A	N/A	N/A
Generic	Were there any other challenges in the healthcare facility?	N/A	N/A	N/A	N/A	N/A

Note: Precision, recall, and F1 are positive-class metrics and are reported only for binary variables. N/A indicates a nonbinary question or a binary variable for which the positive-class metric cannot be estimated because no positive cases were observed.

Supplemental Table 3. Example of prompts for large language model extraction of specific variables related to delays in the "Three Delays-in-Healthcare" framework

Prompt text and required output format	Example questions	Purpose and interpretation
Read the following text about this case.<br>{{text}}<br><br>Based on the text for this case, answer the question and explain why: {{question}}.<br>Answer in 1-2 sentences. Your answer should be formatted as "Yes, ..." or "No, ...".	-Were any resuscitation efforts made for the baby after delivery?<br>-Did the baby die during the initial resuscitation process?	Used for yes/no extraction questions. The model was asked to determine whether the requested event, condition, delay, or care process was documented in the supplied case text and to briefly explain the answer.
Read the following text.<br>{{text}}<br><br>Please note: ANC (antenatal care) visits do not count as clinic visits or illness.<br>Based on the text, answer the question and explain why: {{question}}.<br>Answer in 1-2 sentences. Your answer should be formatted as "Yes, ..." or "No, ...".	-Did mother have any antenatal care visits during this pregnancy?<br>-Did the mother have any antenatal care visits in the first trimester of this pregnancy?<br>-Did the mother have any antenatal care visits in the second trimester of this pregnancy?<br>-Did the mother have any antenatal care visits in the third trimester of this pregnancy?	Used for selected maternal illness and pregnancy-care questions where routine antenatal care visits could be confused with clinic visits for illness. The added instruction clarified that ANC visits should not be counted as illness or illness-related clinic visits.
Read the following text.<br>{{text}}<br><br>Based on the text, answer the question and explain why: {{question}}.<br>Answer in 1-2 sentences. Your answer should be formatted as "Number=..".	-How many antenatal care visits were documented for this pregnancy?	Used for numeric extraction questions, including number of antenatal care visits, gestational timing when pregnancy was recognized, and elapsed time before seeking care after recognizing illness.

Supplemental Table 4. Proportion of stillbirths that experienced each delay in the "Three Delays-in-Healthcare" framework by site, sex, and site of death (N=3,424)

	**Deaths**	**Delay 1**		**Delay 2**		**Delay 3**		**Any delay**	
	**N**	**n (%)**	***P* value***	**n (%)**	***P* value**	**n (%)**	***P* value**	**n (%)**	***P* value**
**Site**			<0.001		<0.001		<0.001		<0.001
Bangladesh	850	170 (20.0)		833 (98.0)		850 (100.0)		850 (100.0)	
Ethiopia	639	4 (0.6)		429 (67.1)		498 (77.9)		575 (90.0)	
Kenya	290	5 (1.7)		172 (59.3)		216 (74.5)		263 (90.7)	
Mali	476	9 (1.9)		465 (97.7)		449 (94.3)		474 (99.6)	
Mozambique	537	37 (6.9)		529 (98.5)		502 (93.5)		533 (99.3)	
Sierra Leone	326	26 (8.0)		223 (68.4)		292 (89.6)		315 (96.6)	
South Africa	306	6 (2.0)		296 (96.7)		245 (80.1)		303 (99.0)	
**Sex (N=3400)**			0.979		0.776		0.872		0.116
Male	1836	137 (7.5)		1574 (85.7)		1639 (89.3)		1786 (97.3)	
Female	1564	118 (7.5)		1352 (86.4)		1391 (88.9)		1503 (96.1)	
**Site of death**			0.005		0.077		0.660		0.872
Community	415	47 (11.3)		343 (82.7)		375 (90.4)		400 (96.4)	
Facility	3009	210 (7.0)		2604 (86.5)		2677 (89.0)		2913 (96.8)	

*P values were calculated using chi-square tests comparing the proportion of deaths with each delay across categories of site, sex, and site of death. Two-sided P<0.05 was considered statistically significant. To account for multiple comparisons, P values were adjusted using the Benjamini-Hochberg false discovery rate method across the 12 omnibus comparisons shown in this table: three grouping variables by four delay outcomes. Adjusted P values are presented

Supplemental Table 5. Proportion of neonatal deaths that experienced each delay in the "Three Delays-in-Healthcare" framework by site, sex, and site of death (N=3,655)

	**Deaths**	**Delay 1**		**Delay 2**		**Delay 3**		**Any delay**	
	**N**	**n (%)**	***P* value***	**n (%)**	***P* value**	**n (%)**	***P* value**	**n (%)**	***P* value**
**Site**			<0.001		<0.001		<0.001		<0.001
Bangladesh	807	806 (99.9)		611 (75.7)		807 (100.0)		807 (100.0)	
Ethiopia	479	247 (51.6)		90 (18.8)		470 (98.1)		473 (98.7)	
Kenya	315	222 (70.5)		48 (15.2)		274 (87.0)		303 (96.2)	
Mali	447	430 (96.2)		277 (62.0)		447 (100.0)		447 (100.0)	
Mozambique	684	614 (89.8)		376 (55.0)		661 (96.6)		678 (99.1)	
Sierra Leone	343	249 (72.6)		80 (23.3)		326 (95.0)		339 (98.8)	
South Africa	580	231 (39.8)		96 (16.6)		497 (85.7)		524 (90.3)	
**Sex (N=3646)**			0.408		0.851		0.851		0.665
Male	2095	1592 (76.0)		910 (43.4)		1999 (95.4)		2051 (97.9)	
Female	1551	1202 (77.5)		666 (42.9)		1477 (95.2)		1513 (97.5)	
**Site of death**			<0.001		<0.001		<0.001		<0.001
Community	680	597 (87.8)		349 (51.3)		675 (99.3)		679 (99.9)	
Facility	2975	2202 (74.0)		1229 (41.3)		2807 (94.4)		2892 (97.2)	

*P values were calculated using chi-square tests comparing the proportion of deaths with each delay across categories of site, sex, and site of death. Two-sided P<0.05 was considered statistically significant. To account for multiple comparisons, P values were adjusted using the Benjamini-Hochberg false discovery rate method across the 12 omnibus comparisons shown in this table: three grouping variables by four delay outcomes. Adjusted P values are presented.

Supplemental Table 6. Proportion of stillbirths and neonatal deaths that experienced each delay in the "Three Delays-in-Healthcare" framework by availability of gestational age or birth weight data

	Deaths	Delay 1		Delay 2		Delay 3		Any delay	
	N	n (%)	*P* value*	n (%)	*P* value	n (%)	*P* value	n (%)	*P* value
				Stillbirths					
**Gestational Age or Birth Weight**			0.029		0.002		0.554		1.000
At least one of gestational age or birth weight available	2,993	1,477 (49.3)		2,560 (85.5)		2,988 (99.8)		2,991 (99.9)	
Both gestational age and birth weight unavailable	431	237 (55.0)		392 (91.0)		430 (99.8)		431 (100.0)	
				Neonatal Deaths					
**Gestational Age or Birth Weight**			0.222		<0.001		0.011		0.023
At least one of gestational age or birth weight available	3,304	2,521 (76.3)		1,388 (42.0)		3,138 (95.0)		3,222 (97.5)	
Both gestational age and birth weight unavailable	351	278 (79.2)		190 (54.1)		344 (98.0)		349 (99.4)	

Pearson chi-square tests were used except when expected cell counts were <5, in which case Fisher's exact test was used.

Supplemental Figure 1. Proportion of included neonatal deaths that experienced each type of delay in the “Three Delays-in-Healthcare” framework by age of neonate at the time of death

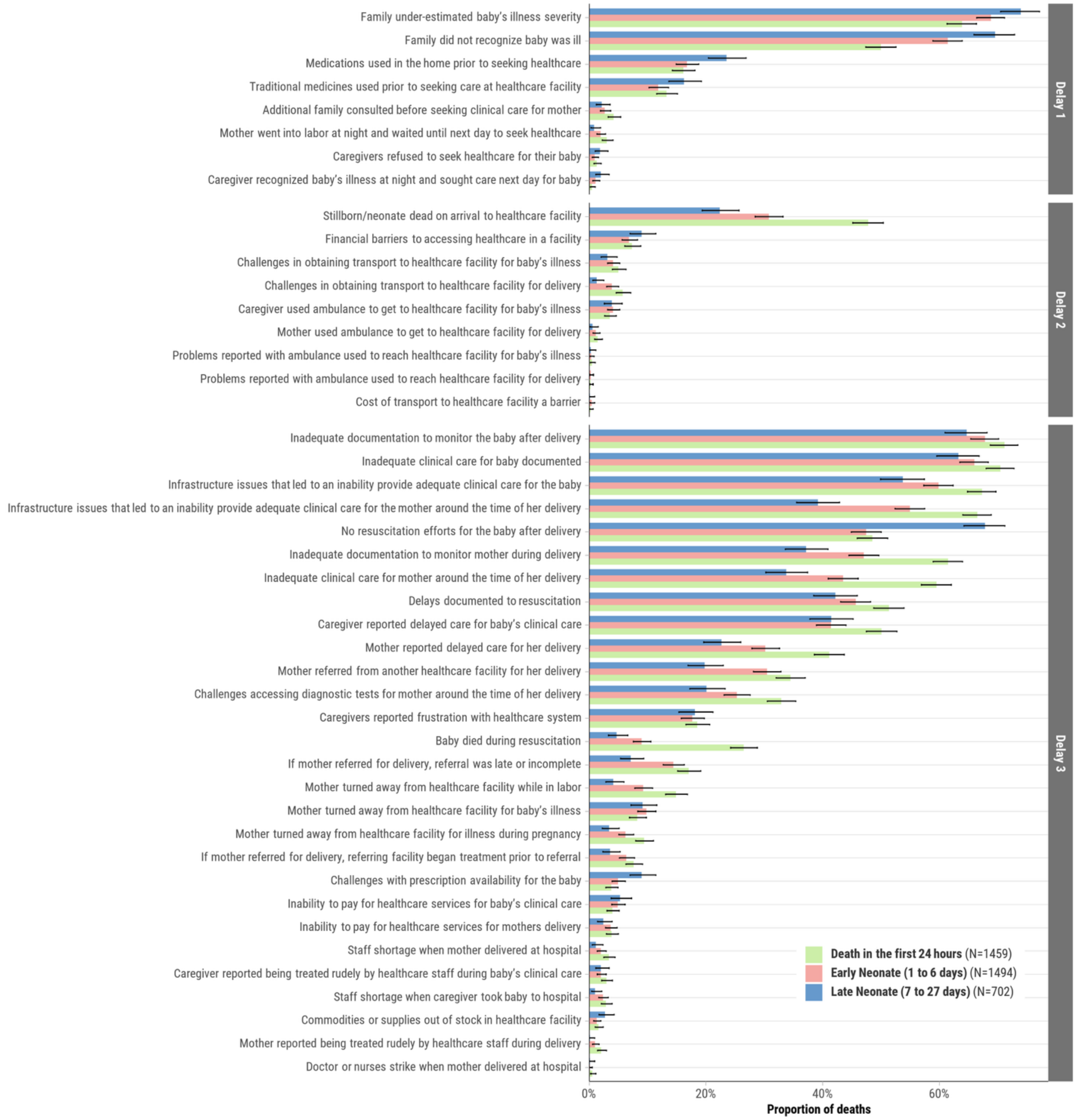

Supplemental Figure 2. Proportions of included cases that experienced each delay over the study period

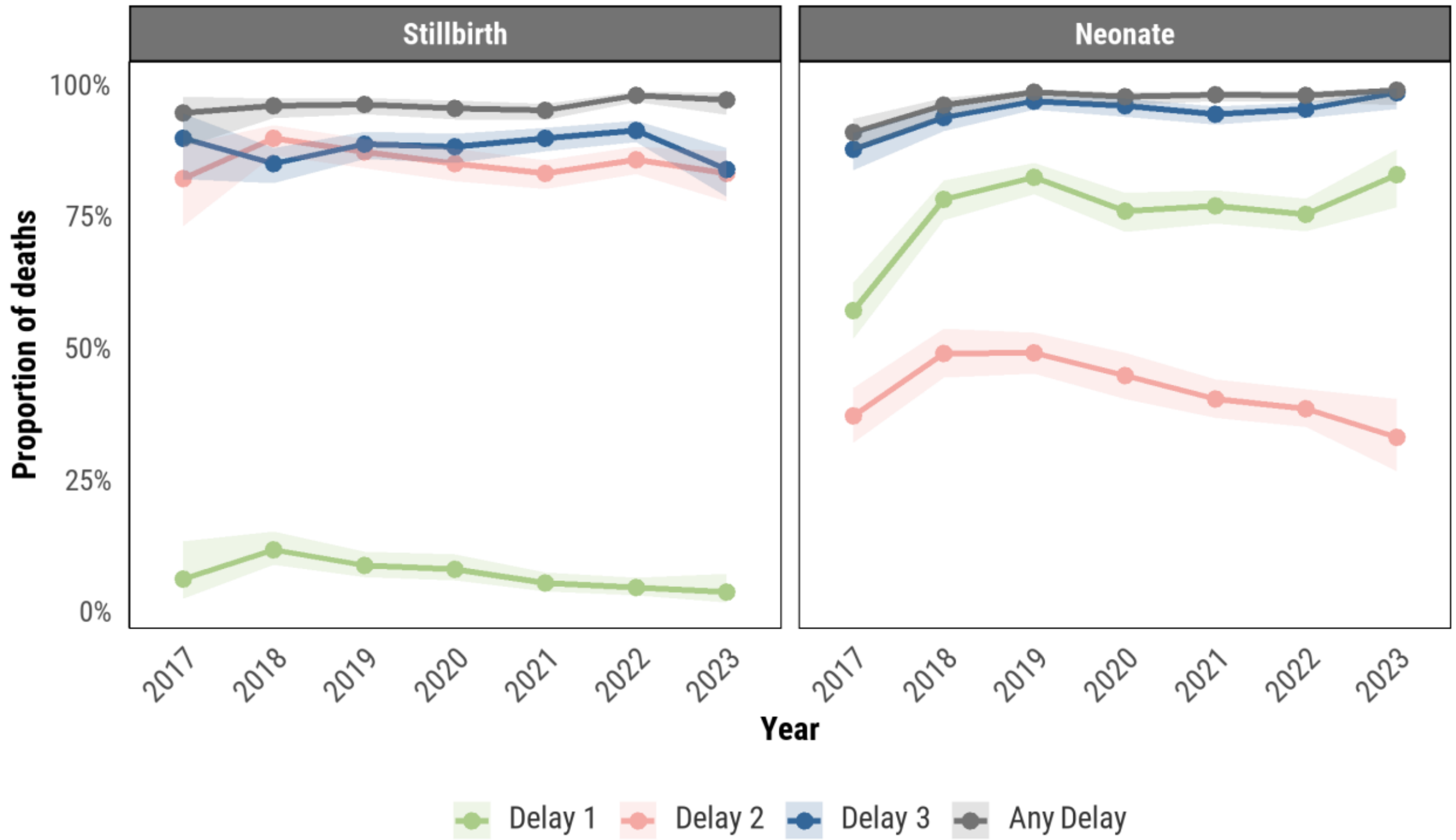

Supplemental Figure 3. Proportion of delays in the "Three Delays-in-Healthcare" framework among deceased a) stillbirths (N=3424 and b) neonatal deaths (N=3655) by country site

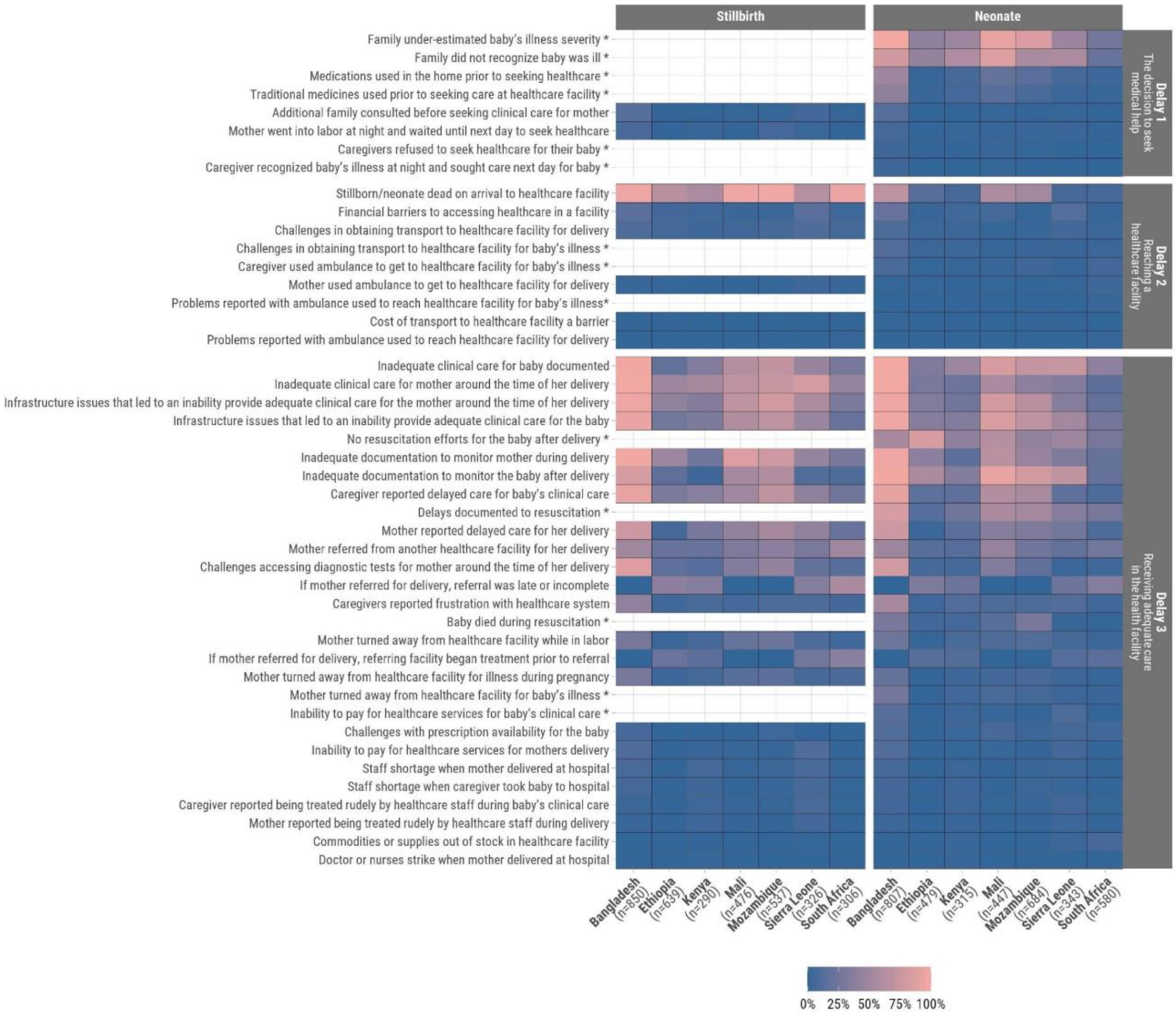

Supplemental Figure 4. Frequencies in delays in the "Three Delays in Healthcare" framework for the leading causes of stillbirths (N=2,115)

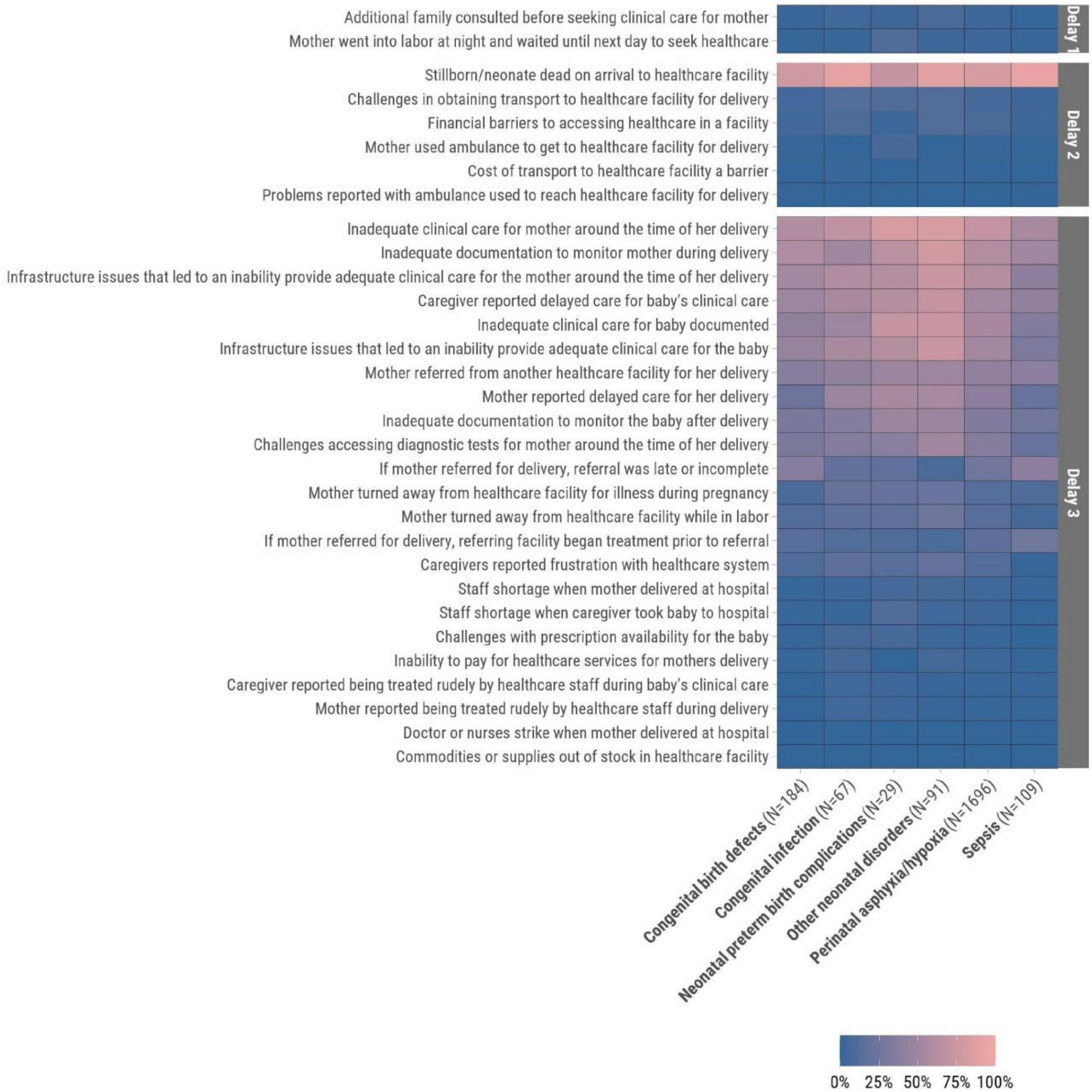

Supplemental Figure 5. Frequencies in delays in the “Three Delays in Healthcare” framework for the leading causes of neonatal deaths (N=2,259)

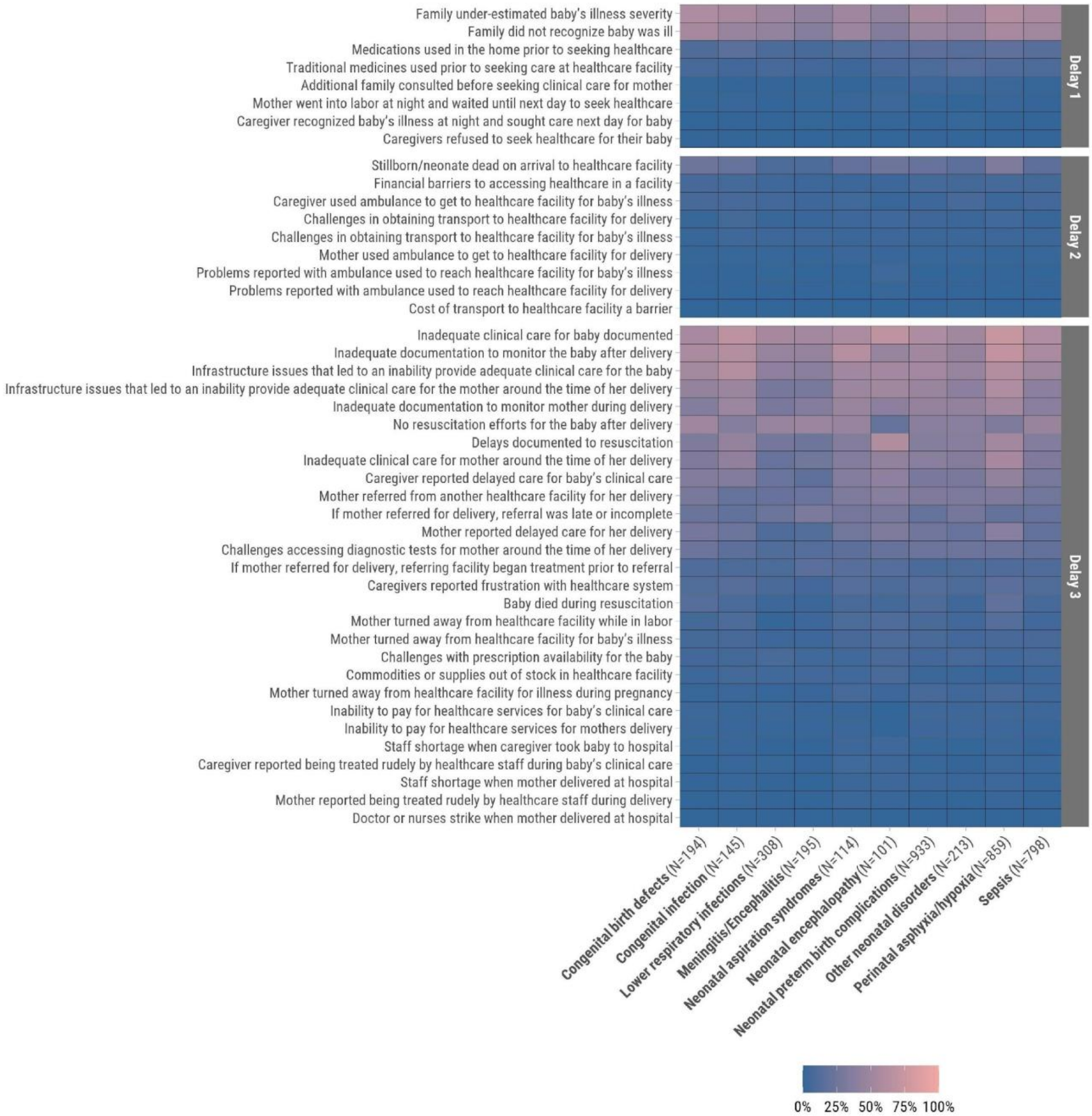

Supplemental Figure 6. Proportion of enrolled cases with antenatal care (ANC) visits documented by CHAMPS site

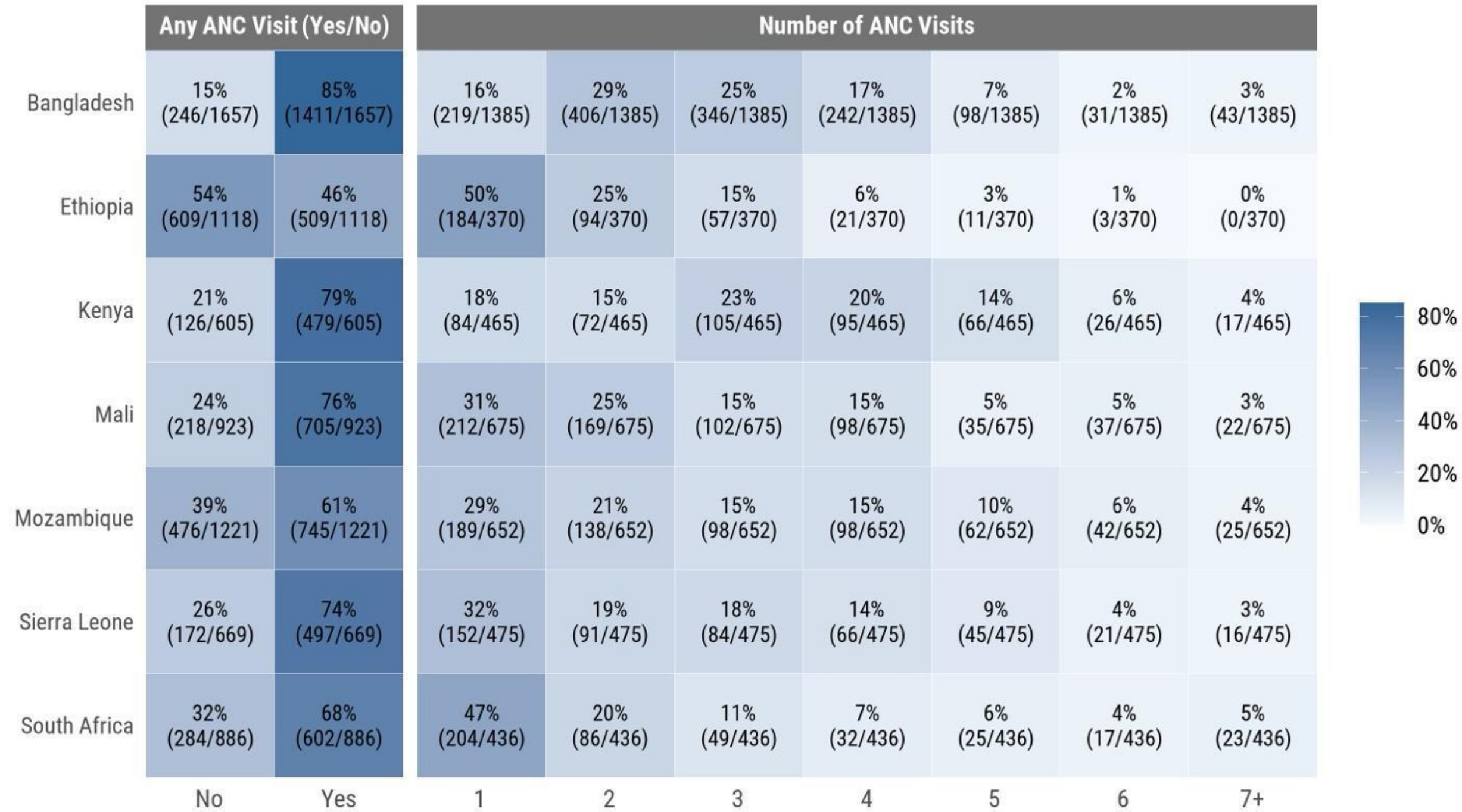

Supplement: Supplementary file 1 [file tpmd260164.SD1.pdf]
